# Childhood onset C3 glomerulopathy: recurrence after kidney transplantation—a case series

**DOI:** 10.3389/fped.2024.1460525

**Published:** 2024-10-21

**Authors:** Yael Borovitz, Daniel Landau, Amit Dagan, Hadas Alfandari, Orly Haskin, Shelly Levi, Gilad Hamdani, Daniella Levy Erez, Shimrit Tzvi-Behr, Jenny Weinbrand-Goichberg, Ana Tobar Foigelman, Ruth Rahamimov

**Affiliations:** ^1^ Nephrology Institute, Schneider Children’s Medical Center, Petah Tikva, Israel; ^2^ School of Medicine, Tel Aviv University, Tel Aviv, Israel; ^3^ Division of Pediatric Nephrology, Shaare Zedek Medical Center, Jerusalem, Israel; ^4^ Department of Pathology, Rabin Medical Center, Beilinson Campus, Petach Tikva, Israel; ^5^ Department of Nephrology and Hypertension, Rabin Medical Center, Beilinson Campus, Petach Tikva, Israel

**Keywords:** C3 glomerulopathy, kidney transplantation, disease recurrence, complement, case series

## Abstract

**Background:**

C3 Glomerulopathy (C3G) is a complement-mediated disease, with predominant C3 deposits, where pathogenic genetic variants in complement system components and circulating autoantibodies result in loss of control of the alternative pathway, have been described. A high incidence of disease recurrence including graft failure has been reported after kidney transplantation (KTx). Currently treatment modalities for preventing and treating post KTx C3G recurrence (plasma exchange, rituximab and eculizumab) in adults have yielded inconsistent results. Data on post KTx C3G recurrence in childhood-onset C3G is still unknown**.**

**Methods:**

A comprehensive case study of patients diagnosed with C3G as children or adolescents, who underwent KTx between the years 2015–2023. Data collected included complement workup, treatment modalities, and outcomes.

**Results:**

19 patients with C3G were identified during the study period. Five patients developed ESRD and received a kidney transplant. C3G recurrence was diagnosed post KTx in 100% of patients. Graft function improved in 3 of these patients (two with anti-factor H antibodies) after eculizumab treatment, one patient reached graft failure 9 months after transplantation despite eculizumab, recieved a second successful transplantation with pre-emptive eculizumab treatment and one patient showed histologic signs of disease recurrence without clinical signs.

**Conclusions:**

C3G recurrence after KTx in patients diagnosed as children or adolescents may be higher than previously described. Treatment with eculizumab is beneficial in some patients. New treatments are needed for improving post-transplant outcome in patients with C3G.

## Introduction

C3 glomerulopathy (C3G) is a complement-mediated disease, with predominant C3 deposits. C3G was re-classified in 2013 and is subdivided to: dense deposit disease (DDD) and C3 glomerulonephritis (C3GN), reflecting kidney biopsy electron microscopy findings (1). According to some published data DDD tends to be a more aggressive disease than C3GN, leading to a higher rate of end stage kidney disease (ESKD) (2, 3). Other reports showed that clinical factors such as kidney function and severity of proteinuria were related to worse long term outcomes (4).

In C3G the pathogenesis is linked to mutations and risk haplotypes in several complement system components and circulating autoantibodies, resulting in the loss of control of the complement system alternative pathway (5). Autoantibodies stabilizing C3 and C5 convertases, including C3 nephritic factor (C3NeF), C5 nephritic factor (C5NeF), factor H mutations or anti-factor H autoantibodies result in a stable convertase, resistant to decay, leading to persistent complement activation. These, together with other mutations and antibodies were described in patients with C3G.

Dysregulation of the complement alternative pathway causes ongoing complement activation, with increased C3 turnover, C3 consumption and systemic low C3 levels. Deposition of complement proteins along the capillary walls of the glomerulus result in mesangial and endocapillary proliferation and capillary-wall remodeling (5, 6).

In adults a high incidence (between 30% and 77%) of C3G disease recurrence has been reported after kidney transplantation (KTx), leading to graft failure in 17%–50% of those affected (7). In a series of 21 transplanted patients with C3GN, diagnosed at a median age of 20.8 years (8), 14 patients (67%) developed recurrence of C3GN in the transplanted kidney at a median time of 28 months from transplantation, and graft failure in 50% of them, at a median time of 77 months from transplantation. Circulating C3 levels prior to transplantation were available for only some of the patients in this cohort, but they were normal in the five patients without recurrence. Other cohorts also reported a high disease recurrence rate, with graft loss in more than 50% of those with disease recurrence (9, 10), which appeared to occur more often in DDD (11).

Therapeutic alternatives of C3G in native kidneys include ACE inhibitors or angiotensin receptor blockers alone in patients with mild disease, to immunosuppression regimens including corticosteroids and mycophenolate mofetil in patients with nephrotic range proteinuria or impaired kidney function (12). In patients with more severe disease or in non-responders to this drug combination, complement inhibitors may be beneficial (13, 14).

Current treatment modalities for C3G recurrence after KTx include removing autoantibodies and repleting factor H using plasma exchange, preventing autoantibodies formation using rituximab, and trying to block terminal complement activation with eculizumab, or other experimental complement blocker agents. The literature is inconsistent regarding the results of these interventions (7, 11, 15, 16).

No guidelines exist regarding the need for prophylactic treatment or conditioning before and after KTx in individuals with C3G. Potential predictive factors for disease recurrence include certain genotypes, the presence of autoantibodies, and the current status of complement dysregulation. Still, no specific data are available to guide decisions prior to transplantation, and current recommendations are based on expert opinion and case reports (8, 17).

Regarding childhood onset C3G and disease recurrence after kidney transplantation—one case report describes pediatric transplant related C3G: a patient with a diagnosis of DDD in the native kidneys and recurrence after transplantation, who was treated with rituximab and eculizumab (15), In this patient the treatment effectively inhibited the terminal complement cascade but only partially prevented disease progression. Other cases of pediatric onset C3G were described along with adult cohorts. The disease recurrence rate after KTx in childhood onset C3G, the risk factors for recurrence, and the optimal treatment of recurrence are still unknown. Therefore, this study aimed to describe a case series of transplanted patients with childhood onset C3G, including treatment modalities and outcomes.

## Methods

For this case series study, data were collected of patients diagnosed with C3G as children or adolescents in 2 large pediatric nephrology units and underwent a KTx between the years 2015–2023. All patients underwent a native kidney biopsy at presentation. A for cause kidney biopsy in the native or transplanted kidney was performed for indication (proteinuria, increase of serum creatinine). All kidney biopsies were interpreted by a nephropathologist. The biopsy interpretation was based on the current definition of C3G including light microscopy findings of glomerulonephritis with predominant C3 deposits and findings of DDD or C3GN in electron microscopy (1).

For all patients, a detailed medical history, as well as clinical, laboratory and histopathological data, were available regarding native kidney and transplanted kidney disease. All patients underwent a functional and genetic complement system workup at the molecular otolaryngology and renal research laboratories in the University of Iowa—including—levels of factor H, factor I, factor D, anti-factor H antibodies, anti-Factor B antibodies, C3Nef, C5Nef, terminal complement activity markers, and other complement biomarkers. Genetic tests for CFH, CFI, MCP, CFB, CFHR5, C3, THBD, DGKE, PLG, ADAMTS13, MMACHC, G6PD, WT1including copy number variation screening of the CFH-CFHR5 region using multiple ligation dependent probe amplification.

Post KTx C3G recurrence was determined according to histology findings of transplanted kidney biopsy showing features of C3G as defined by Pickering et al. C3 Glomerulopathy: consensus report (1). The study was approved by the local Helsinki committee and the Israel Ministry of Health.

## Results

During the study period, out of 19 patients with C3G who were actively followed, five reached ESKD and underwent KTx. Patients’ characteristics are described in Table 1. Initial C3G diagnosis median age was 16 years (range: 7.5–17). According to biopsy at presentation, three patients had features consistent with DDD. Electron microscopy was not available for the other two patients.

**Table 1:** Characteristics of the patients.

	Patient 1		Pateint 2	Pateint 3	Pateint 4	Pateint 5
Gender	F		M	F	M	M
Age at presentation (years)	7.5		15	12	17	16
Native kidney biopsy	C3G		DDD	DDD	DDD	C3G
EM available	No		Yes	Yes	Yes	No
Treatment for native kidney disease	CS, CYC		CS, CYC, MMF	CS, CYC, MMF, PP, RTX	CS, CYC, MMF, CsA	None
Time to ESKD (years)	0		2	8	13	0
KT #	1	2	1	1	1	1
Age at KT (years)	9	12	17	20	30	16.5
Time from KT to recurrence (months)	2	No recurrence	2	18	8	2
Clinical/laboratory signs of disease recurrence	H, P, elevated SCr	none	none	H, P, elevated SCr	P, Hi SCr	P, elevated SCr
Transplanted kidney biopsy	DDD	Not preformed	DDD	DDD	DDD	C3G (EM unavailable)
Treatment of recurrence	ECU	Prophylactic ECU	none	PP, RTX, ECU	RTX, ECU	CS, ECU
Post KT follow up (months)	45	12	45	43	26	11
SCr at last follow up (mg/dl)	On dialysis	0.56	1.3	1.26	1.19	1.9
UPCR at last follow up (mg/mg)	anuria	0.2	0.13	0.26	0.9	0.16
Highest UPCR (mg/mg)	11.4	0.2	0.25	9.6	4.8	0.57

DDD, dense deposit disease; CS, Corticosteroids; CYC, Cyclophosphamide; MMF, Mycophenolate Mofetil; PP, Plasmapheresis; CsA, Cyclosporin A; ESKD, end stage kidney disease; KT, Kidney transplantation; H,hematuria; P, proteinuria; SCr, serum creatinine; UPCR, urine protein/creatinine ratio.

Four patients were initially treated with immunosuppression for their native kidney disease, including corticosteroids, cyclophosphamide and mycophenolate mofetil in 3 patients and plasmapheresis in one patient. In spite of that, all reached ESKD. Two patients presented with dialysis dependent kidney failure, one did not respond to immunosuppressive treatment and in the other one treatment was not initiated due to diffuse global sclerosis in the native kidney biopsy. The mean time from the initial diagnosis to ESKD was 4.6 (range: 0–17) years.

All patients had low C3 levels at initial presentation. Two patients were positive for anti factor H antibodies (titers of 3,087 AU and 1960AU, normal range <200 AU), one of them was also positive for both C3Nef and C5Nef. Another patient was positive for both C3Nef and C5Nef, one patient had low Factor B levels (Table 2). Genetic tests were positive for a pathogenic variant only in one patient with factor H antibodies who had a homozygous deletion of the CFHR3-CFHR1 gene. Two other patients had findings described as variants of unknown significance, and one of them had also CFH gene associated risk alleles (Table 2).

**Table 2:** Complement system biomarkers and genetic analysis.

	Patient 1	Patient 2	Patient 3	Patient 4	Patient 5
C3 at presentation (mg/dl)^a^	20	62.8	6.6	8.8	19
C3 at KT (mg/dl)^a^	1st KT: 9.3	75.7	10.6	7.6	12.6
	2nd KT: 13.8				
C3 at recurrence (mg/dl)	30	92	7.4	NA	63
C3 at last follow up (mg/dl)^a^	1st KT 21.7	91	9.2	6.6	76
	2nd KT 25				
Complement workup	C3Nef	Low factor B level	Anti factor H antibodies	Anti factor H antibodies	Borderline C3Nef
	C5Nef			C3Nef	
				C5Nef	
Genetic tests	Multiple VUS^b^ Risk alleles—3 copies of *CFH*- associated risk alleles	Negative	Homozygous deletion CFHR3-CFHR1	Negative	Negative

NA, not available; C3Nef, C3 nephritic factor; C5Nef, C5 nephritic factor; VUS, variant of unknown significance.

^a^ C3- Normal range, 90–180 mg/dl.

^b^ C8B, CFH, CFHR5, FCN1, PLG.

**Figure 1:**
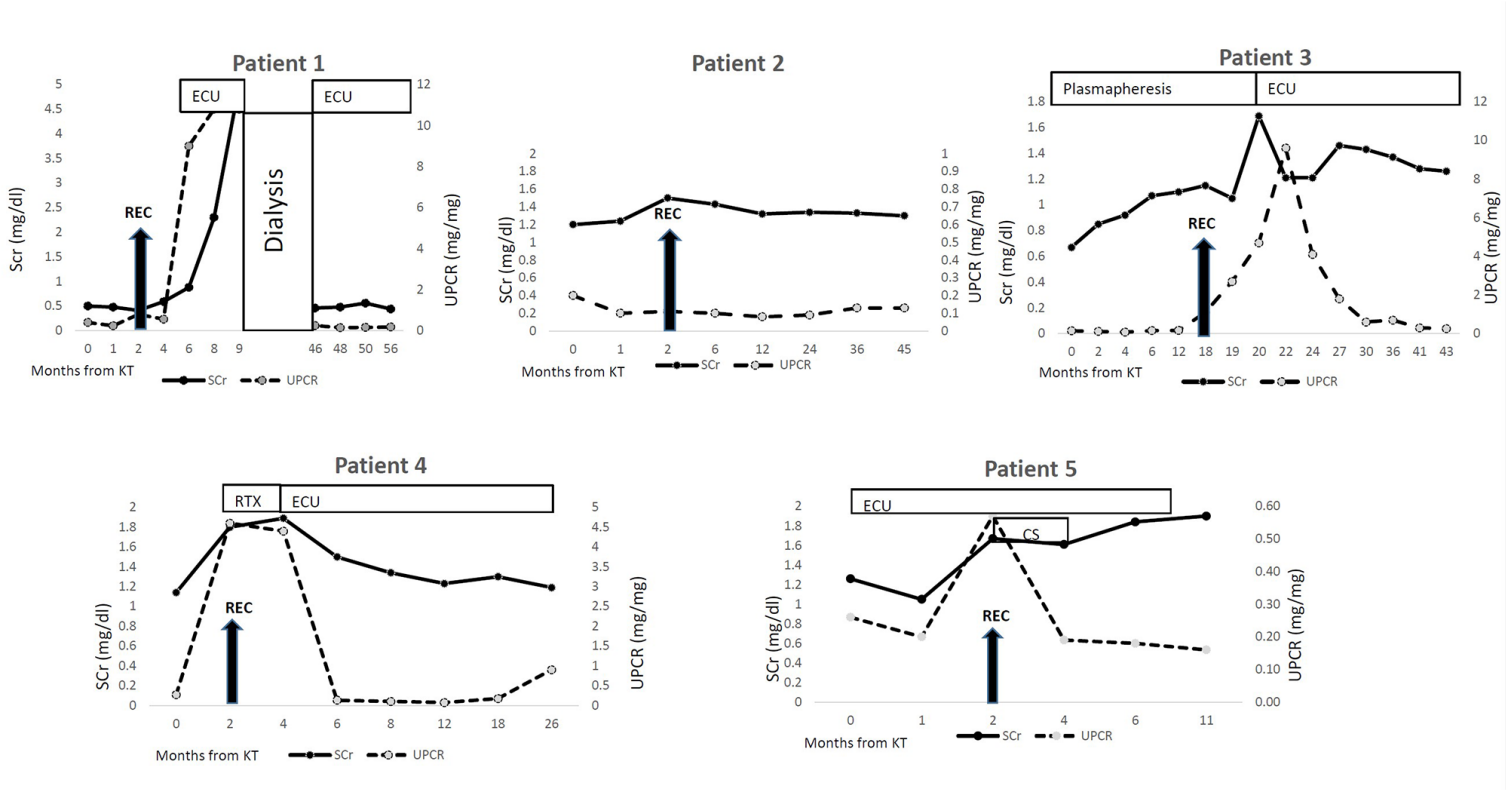
Disease course after kidney transplantation. KT- Kidney transplantation REC- recurrence CS- corticosteroids RTX- rituximab ECU-eculizumab SCr- serum creatinine UPCR- urine protein/creatinine ratio.

All patients underwent KTx, one of them recieved 2 deceased donors KTx's during the study period. All other KTx's were from living non-related donors. The median age at first KTx was 17 (range 9–30) years. All patients remained with low C3 levels at the time of transplantation (as shown in Table 2). Disease course after KT reflected by creatinine and proteinuria values is shown in Figure 1.

C3G transplanted patients were treated according to local protocol including induction therapy using Basiliximab (for the first transplantation) and anti thymocyt globulin (ATG) for second transplantation, and triple immunosuppression regimen including tacrolimus, mycophenolate mofetil and corticosteroids. Maintenance immunosuppression included tacrolimus (trough level 5–7 ng/ml), mycophenolate mofetil in all patients and low dose corticosteroids. One patient who was known to have anti factor H antibodies (patient 3) was treated with plasmapheresis and Rituximab prior to and after KTx, before recurrence was diagnosed. Patient 4 who showed signs of complement dysregulation on immunologic workup– received Eculizumab post transplantation. All patients had a recurrence of C3G in the transplanted kidney. Pathological findings in all the instances of recurrence were consistent with DDD. Immunofluorescence staining was positive for C3 deposits in all the patients. Patient 2 had only histologic signs of C3G recurrence but no clinical signs. His biopsies were performed two and five months after KTx due to mild T-cell mediated rejection and polyoma BK nephropathy, respectively. Both biopsies showed C3 deposits by immunofluorescence and electron dense deposits in electron microscopy.

Four patients had clinical signs of disease recurrence including microscopic hematuria, proteinuria, and elevation of serum creatinine. Patient 1, the youngest in the cohort, had severe and rapid disease recurrence which did not respond to eculizumab treatment, and reached graft failure within 9 months after first transplantation. Almost 3 years after her first KTx, she had a second KTx with prophylactic eculizumab treatment, with no signs of recurrence 12 months after. This patient was positive for C3Nef and C5Nef showing very high titers before 1st and 2nd transplantation (77%, 73% and 79%, 91% respectively (normal range <20%).

Patient 3, who was positive for anti-factor H antibodies, was treated before and after transplantation with weekly plasmapheresis and rituximab. Recurrence was observed 18 months after transplantation and treatment was switched to eculizumab. Patient 4, who was also positive for factor H antibodies, had signs of disease recurrence 8 months after transplantation. He was initially treated with rituximab which did not impact the proteinuria and treatment with eculizumab was initiated. Eculizumab treatment for both these patients was beneficial: proteinuria improved and kidney function normalized. Patient 3 reached full remission with no proteinuria or hematuria, patient 4 had normal serum creatinine and mild proteinuria at last follow up (16 months after treatment initiation).

Patient 5 was treated with Eculizumab since transplantation due to evidence of ongoing complement dysregulation. Two months after transplantation, there was evidence of disease recurrence. He received corticosteroid pulse therapy and continued eculizumab treatment, with normalization of proteinuria and improvement in serum creatinine level. Five months after KTx a second biopsy was preformed due to creatinine elevation—showing chronic transplant glomerulopathy, with no evidence of C3G recurrence.

## Discussion

Recurrence of complement mediated kidney disease after KTx is a major concern and a main cause for graft loss. Complement dysregulation can be due to congenital or acquired causes. Like other complement mediated kidney diseases, C3G has a high rate of recurrence after KTx, our experience showed a 100% disease recurrence rate. For other diseases, such as atypical hemolytic uremic syndrome, prophylactic treatment with complement C5 inhibitor (eculizumab) prevents disease recurrence in most patients and dramatically changed outcomes of KTx (18). In contrast, no treatment has been shown to consistently prevent recurrence of C3G. In cases of C3G the mechanism of injury is different from atypical HUS as depositions of C3 split products in the mesangium initiates the inflammatory process. Case reports describe successful treatment with eculizumab in some patients (15, 19, 20), while others report eculizumab failure in preventing C3G recurrence (21). Failure of eculizumab treatment (which prevents C5 activation) may be associated with the presence of C3 nephritic factor, which leads to a predominant C3 (proximal to C5) activation. Analyzing C3Nef-mediated C3 and C5 activation separately could help in selecting the appropriate patients for eculizumab therapy. In our small cohort eculizumab was effective in three out of 5 cases of C3G recurrence, two of them with anti factor H antibodies. Factor H is a key inhibitor of complement overactivation, and mutations or antibodies in this protein lead to atypical HUS or C3GN.

Other treatments such as plasma exchange and rituximab have shown various results and success rates, and may be related to the primary reason for complement dysregulation in each patient (7). For patients who are positive for anti-factor H autoantibodies, these antibody depleting treatments may be reasonable, but from our experience, the patients who were positive for anti factor H antibodies did not reach remission under this treatment, and improved only after changing treatment to eculizumab.

Among the adult population, recurrence rates are estimated as 30%–70%. In a summary of small series and case reports which included a total of 122 patients with post-transplant C3G (73 C3GN and 49 DDD) (7), the authors reported higher allograft loss with plasmapheresis and rituximab compared to eculizumab. Subgroup analysis showed a higher rate of graft loss despite treatment among patients with DDD compared with C3GN.

Recurrence rates among patients who presented with C3G as children or adolescents are still unknown. Our cohort exhibited a recurrence rate of 100%. A suggested explanation might be that the disease penetrance is higher in children leading to an early presentation in childhood and a more aggressive form of disease leading to higher rates of recurrence following transplant. Additional research is needed to understand these differences. Recurrence was symptomatic in four of the five patients with recurrence. One of them, the youngest of the cohort, showed rapid deterioration to ESKD and dialysis. While undergoing dialysis treatments, C3 levels remained very low and repeat complement workup still showed ongoing complement dysregulation, and presence of C3 and C5 nephritic factors. This patient had a second KTx using prophylactic eculizumab treatment with no signs of recurrence during 12 months of follow up. The other two patients who were positive for factor H antibodies also had disease recurrence, which was controlled under eculizumab treatment, similar to previous reported patients (22). Previous studies described few cases of second transplantation after C3G recurrence with various outcomes (11), to our opinion in such cases prophylactic complement blocker treatment should be considered.

The question of to how to address low C3 levels before transplantation remains unanswered. Low C3 levels may indicate ongoing complement activation, but C3 levels do not properly correlate with disease activity: 25% of pediatric C3G patients and about 50% of adult C3G patients present with normal blood C3 levels despite an active disease (23). Measurement of circulating terminal attack complex (C5b-9) levels prior to transplantation, may aid the decision regarding prophylactic treatment as patient with elevated circulating C5b-9 may benefit from eculizumab treatment. In our cohort four patients were treated successfully with eculizumab two had high C5b-9 levels one had normal C5b-9 level and for one C5b-9 level before treatment initiation was unavailable.

In patients with C3G, heterogeneity is observed in the exact underlying complement disorders that cause complement dysregulation and ongoing activation. Understanding the particular complement disorder may aid in risk stratification for disease recurrence. The novel complement system blockers that are currently examined in clinical trials may offer a new horizon for individuals with C3G who undergo KTx (16, 24, 25). A better understanding of the underlying complement disorders will contribute to decision making, specifically, the optimal prophylactic treatment to prevent disease recurrence, if any, for each patient.

Our study has several limitations. First—this is a single center study dealing with a rare complement mediated disease—therefore the number of patients is very small. The second limitation is that complement biomarkers testing is only available only and in a few laboratories around the world—so real time and repeat tests cannot be performed. Third—in our center we do not conduct protocol biopsies—therefore we cannot know at this point whether patient 1 is experiencing subclinical recurrence in the second graft.

Despite the small cohort there are lessons learned from this study—C3G is a heterogeneous disease, and time from presentation to ESKD is variable. Disease recurrence can be asymptomatic and may not need any additional treatment. In cases of symptomatic recurrence or in cases of second transplantation eculizumab or other experimental complement blocking agent may serve as a potential treatment.

## Conclusion

C3G recurrence rate after KTx in patients diagnosed as children or adolescents may be higher than described in the adult onset C3G. Treatment with currently available tools is successful in only some patients. New treatments are needed for safe transplantation in the context of C3G and for avoidance of disease recurrence.

## Data Availability

The raw data supporting the conclusions of this article will be made available by the authors, without undue reservation.

## Summary at glace

Patients diagnosed with C3 Glomerulopathy as children or adolescents were followed after kidney transplantation. All patients developed disease recurrence after transplantation confirmed by kidney biopsy. Some responded to therapy with improved proteinuria and renal function.

## References

[1] Pickering MC D'Agati VD Nester CM Smith RJ Haas M Appel GB C3 glomerulopathy: consensus report. Kidney Int. (2013) 84(6):1079–89. 10.1038/ki.2013.377 24172683 PMC3842953

[2] Ravindran A Fervenza FC Smith RJH De Vriese AS Sethi S. C3 glomerulopathy: ten years’ experience at mayo clinic. Mayo Clin Proc. (2018) 93(8):991–1008. 10.1016/j.mayocp.2018.05.019 30077216 PMC6312642

[3] Michels MAHM Wijnsma KL Kurvers RAJ Westra D Schreuder MF van Wijk JAE Long-term follow-up including extensive complement analysis of a pediatric C3 glomerulopathy cohort. Pediatr Nephrol. (2022) 37(3):601–12. 10.1007/s00467-021-05221-6 34476601 PMC8921070

[4] Pınarbaşı AS Dursun I Gokce I Çomak E Saygılı S Bayram MT Predictors of poor kidney outcome in children with C3 glomerulopathy. Pediatr Nephrol. (2021) 36(5):1195–205. 10.1007/s00467-020-04799-7 33130981

[5] Riedl M Thorner P Licht C. C3 glomerulopathy. Pediatr Nephrol. (2017) 32(1):43–57. 10.1007/s00467-015-3310-4 27056062

[6] Smith RJH Appel GB Blom AM Cook HT D'Agati VD Fakhouri F C3 glomerulopathy — understanding a rare complement-driven renal disease. Nat Rev Nephrol. (2019) 15(3):129–43. 10.1038/s41581-018-0107-2 30692664 PMC6876298

[7] Suarez ML G Thongprayoon C Hansrivijit P Kovvuru K Kanduri SR Aeddula NR Treatment of C3 glomerulopathy in adult kidney transplant recipients: a systematic review. Med Sci (Basel). (2020) 8(4):44. 10.3390/medsci8040044 33096866 PMC7712822

[8] Zand L Lorenz EC Cosio FG Fervenza FC Nasr SH Gandhi MJ Clinical findings, pathology, and outcomes of C3GN after kidney transplantation. J Am Soc Nephrol. (2014) 25(5):1110–7. 10.1681/ASN.2013070715 24357668 PMC4005307

[9] Kumar A Ramachandran R Rawat A Das R Rayat CS Kenwar DB Poor allograft outcome in Indian patients with post-transplant C3 glomerulopathy. Clin Kidney J. (2021) 14(1):291–300. 10.1093/ckj/sfz135 33564431 PMC7857824

[10] Lu DF Moon M Lanning LD McCarthy AM Smith RJ. Clinical features and outcomes of 98 children and adults with dense deposit disease. Pediatr Nephrol. (2012) 27(5):773–81. 10.1007/s00467-011-2059-7 22105967 PMC4423603

[11] Regunathan-Shenk R Avasare RS Ahn W Canetta PA Cohen DJ Appel GB Kidney transplantation in C3 glomerulopathy: a case series. Am J Kidney Dis. (2019) 73(3):316–23. 10.1053/j.ajkd.2018.09.002 30413277

[12] Avasare RS Canetta PA Bomback AS Marasa M Caliskan Y Ozluk Y Mycophenolate Mofetil in Combination with Steroids for Treatment of C3 Glomerulopathy. Clin J Am Soc Nephrol. (2018) 13(3):406–13. 10.2215/CJN.09080817 29326307 PMC5967675

[13] Bomback AS Smith RJ Barile GR Zhang Y Heher EC Herlitz L Eculizumab for dense deposit disease and C3 glomerulonephritis. Clin J Am Soc Nephrol. (2012) 7:748–56. 10.2215/CJN.12901211 22403278 PMC3338285

[14] Le Quintrec M Lionet A Kandel C Bourdon F Gnemmi V Colombat M Eculizumab for treatment of rapidly progressive C3 glomerulopathy. Am J Kidney Dis. (2015) 65:484–9. 10.1053/j.ajkd.2014.09.025 25530108

[15] Gurkan S Fyfe B Weiss L Xiao X Zhang Y Smith RJ. Eculizumab and recurrent C3 glomerulonephritis. Pediatr Nephrol. (2013) 28:1975–81. 10.1007/s00467-013-2503-y 23689905 PMC4428658

[16] Wong E Nester C Cavero T Karras A Le Quintrec M Lightstone L Efficacy and safety of iptacopan in patients with C3 glomerulopathy. Kidney Int Rep. (2023) 8(12):2754–64. 10.1016/j.ekir.2023.09.017 38106570 PMC10719607

[17] Goodship TH Cook HT Fakhouri F Fervenza FC Frémeaux-Bacchi V Kavanagh D Atypical hemolytic uremic syndrome and C3 glomerulopathy: conclusions from a “kidney disease: improving global outcomes” (KDIGO) controversies conference. Kidney Int. (2017) 91(3):539–51. 10.1016/j.kint.2016.10.005 27989322

[18] Zuber J Frimat M Caillard S Kamar N Gatault P Petitprez F Use of highly individualized complement blockade has revolutionized clinical outcomes after kidney transplantation and renal epidemiology of atypical hemolytic uremic syndrome. J Am Soc Nephrol. (2019) 30(12):2449–63. 10.1681/ASN.2019040331 31575699 PMC6900783

[19] Sahin H Gok Oguz E Akoglu H Atilgan G Ulusal Okyay G Karaveli Gursoy G Successful treatment of posttransplant recurrent complement C3 glomerulopathy with eculizumab. Iran J Kidney Dis. (2018) 12(5):315–8. 30367025

[20] McCaughan JA O'Rourke DM Courtney AE. Recurrent dense deposit disease after renal transplantation: an emerging role for complementary therapies. Am J Transplant. (2012) 12(4):1046–51. 10.1111/j.1600-6143.2011.03923.x 22233157

[21] Kaartinen K Martola L Räisänen-Sokolowski A Meri S. Recurrent allograft C3 glomerulonephritis and unsuccessful eculizumab treatment. Clin Immunol. (2018) 187:104–6. 10.1016/j.clim.2017.10.013 29097196

[22] Garg N Zhang Y Nicholson-Weller A Khankin EV Borsa NG Meyer NC C3 glomerulonephritis secondary to mutations in factors H and I: rapid recurrence in deceased donor kidney transplant effectively treated with eculizumab. Nephrol Dial Transplant. (2018) 33(12):2260–5. 10.1093/ndt/gfx369 29370420 PMC6275145

[23] Heiderscheit AK Hauer JJ Smith RJH. C3 glomerulopathy: understanding an ultra-rare complement-mediated renal disease. Am J Med Genet C Semin Med Genet. (2022) 190(3):344–57. 10.1002/ajmg.c.31986 35734939 PMC9613507

[24] Schubart A Anderson K Mainolfi N Sellner H Ehara T Adams CM Small-molecule factor B inhibitor for the treatment of complement-mediated diseases. Proc Natl Acad Sci U S A. (2019) 116(16):7926–31. 10.1073/pnas.1820892116 30926668 PMC6475383

[25] Hoy SM. Pegcetacoplan: first approval. Drugs. (2021) 81(12):1423–30. 10.1007/s40265-021-01560-8 34342834

